# Collaborative dietetic and psychological care in Interprofessional Enhanced Cognitive Behaviour Therapy for adults with Anorexia Nervosa: a novel treatment approach

**DOI:** 10.1186/s40337-023-00743-w

**Published:** 2023-02-27

**Authors:** Megan Bray, Gabriella Heruc, Susan Byrne, Olivia R. L. Wright

**Affiliations:** 1 School of Human Movement and Nutrition Sciences, The University of Queensland, Brisbane, Australia; 2 Eating Disorders and Nutrition Research Group (ENRG), School of Medicine, Western Sydney University, Penrith, Australia; 3 The Swan Centre, Claremont, Australia

**Keywords:** Anorexia nervosa, Dietitians, Eating disorders, Interprofessional collaboration, Interdisciplinary health team, Malnutrition, Multidisciplinary care, Nutritional management, Patient care team, Psychologist, Mental health professional

## Abstract

Clinical practice guidelines for anorexia nervosa (AN) and atypical anorexia nervosa recommend treatment from a team including a mental health professional and a dietitian. To date, however, AN treatment models such as Enhanced Cognitive Behaviour Therapy (CBT-E) seldom include dietitians and have low to moderate treatment efficacy. Given interprofessional approaches to healthcare have been shown to improve treatment outcomes and enhance patient and clinician satisfaction, formalising collaborative dietetic and psychological treatment may be a feasible strategy to improve treatment outcomes and the patient and clinician experience of treatment. Moreover, malnutrition is a serious consequence of AN, and dietitians are considered experts in its diagnosis and treatment. This paper proposes a novel treatment approach, Interprofessional Enhanced Cognitive Behaviour Therapy (CBT-IE), an adaptation of CBT-E where dietitians deliver content related to malnutrition and dietary restraint and mental health professionals deliver content related to cognitive and behavioural change. The rationale for developing CBT-IE, treatment structure, and future research directions is discussed.

**Supplementary Information:**

The online version contains supplementary material available at 10.1186/s40337-023-00743-w.

## Introduction

Anorexia nervosa (AN) and atypical anorexia nervosa (hereafter referred to as AN) are psychiatric disorders with severe physiological and psychosocial consequences. To comprehensively treat AN, recent clinical practice guidelines advise integrated treatment from a team including a mental health professional and dietitian [1, 2]. Despite recommendations, collaborative treatment between mental health professionals and dietitians is poorly defined in eating disorder treatment models, which are often led by one health professional. Moreover, there is limited evidence supporting dietetic involvement and interprofessional treatment for eating disorders.

Leading eating disorder treatment models, such as Enhanced Cognitive Behaviour Therapy (CBT-E), and Maudsley Anorexia Treatment for Adults (MANTRA), were designed to be led by a mental health professional and the current treatment manuals do not describe how dietetic treatment could be integrated [3, 5]. Specialist Supportive Clinical Management, however, has recently been revised to offer dietetic treatment in a flexible manner responsive to individual needs [5]. This incongruency between practice guidelines and therapy models creates uncertainty regarding collaborative AN treatment between mental health professionals and dietitians and may lead to discrepant approaches in clinical practice. To address this gap and provide evidence of the efficacy of interprofessional treatment involving a dietitian, we propose an adaptation to CBT-E where the dietitian and mental health professional deliver treatment collaboratively in a manner reflecting their expertise.

Collaborative team approaches to healthcare are considered essential, with health policy makers worldwide endorsing Interprofessional Collaborative Practice (ICP) as the modern benchmark [6]. ICP is characterised by common goals, mutual respect, shared values, clear roles and responsibilities, interprofessional communication and principles of teamwork [7]. Implementation of ICP is complex, spanning policy, community, organisational and individual client-practitioner levels [8, 9]. In the mental health sector, ICP is associated with improved health outcomes, reduced treatment dropout, increased patient satisfaction with services and greater work-related satisfaction among clinicians [6]. Given current treatment for AN is often associated with low to moderate efficacy [10, 11], marked treatment dropout [12] and high levels of burnout among clinicians [13], ICP between mental health professionals and dietitians in AN treatment may have benefits for individuals experiencing an eating disorder as well as for clinicians.

CBT-E addresses the maintaining mechanisms of eating disorders using cognitive and behavioural strategies [4]. Despite its standing as a leading treatment approach, CBT-E is effective for only 30–60% of individuals with AN [14] with reported dropout rates ranging from 20 to 50% [15, 16]. Therefore, adaptations may be warranted. Since the model recognises dietary restriction and low body weight, two factors related to malnutrition, and dietary restraint, as core maintaining features of AN, an interprofessional approach involving a dietitian may be beneficial. Moreover, as research has not supported stand-alone dietetic treatment intervention [17], development of interprofessional treatment approaches involving dietitians is essential to guide practice.

Incorporating a dietitian in CBT-E for AN may be of merit due to dietitians’ qualifications in malnutrition treatment. Malnutrition is present in the context of two of more of the following conditions: insufficient energy intake, weight loss, loss of muscle mass, loss of subcutaneous fat, localised or generalised fluid accumulation and diminished functional status [18]. In AN, given malnutrition is a primary consequence of restrictive eating and compensatory behaviours [19], and it contributes to increased morbidity and mortality, decreased function and quality of life, protracted hospital admissions and higher healthcare costs [18], appropriate management is critical. Treatment of malnutrition requires tailored nutritional intervention from a specialist professional to provide increased energy, macronutrients and micronutrients relevant to individual requirements [20]. Dietitians are recognised as highly qualified professionals when it comes to malnutrition diagnosis and treatment due to their training in physiology, biochemistry, nutrition counselling and Medical Nutrition Therapy [21]. Additionally, recent practice standards for dietitians treating eating disorders promote ongoing training and supervision in this practice area [22]. Therefore, involving dietitians in the collaborative treatment of AN might ensure malnutrition is most effectively managed, resulting in earlier nutritional rehabilitation and improved treatment outcomes.

Engaging a dietitian in CBT-E also supports a comprehensive assessment of the patient’s nutritional status and delivery of nutrition interventions which optimise physical state [23]. In current AN treatment, return to physical health is commonly measured by weight restoration to a ‘normal’ body mass index (BMI) [4], often with limited focus on more nuanced aspects of physical health; including body composition, individual biochemistry, clinical symptoms related to the cardiovascular, endocrine, musculoskeletal and gastrointestinal systems and diet quality [24]. This approach may limit nutritional rehabilitation to introducing regular eating, using calorie dense foods and supplements and conducting behavioural experiments with feared foods [4], rather than individualising dietary advice to meet energy, macronutrient and micronutrient requirements in order to address malnutrition and nutritional deficiencies [25]. A tailored approach may be especially pertinent when the weight of an individual with AN is within a ‘normal,’ ‘overweight,’ or ‘obese’ range and BMI may not accurately depict health status [26]. The inclusion of a dietitian in treatment of AN goes beyond using BMI as a primary marker of physiological wellbeing and promotes consideration of a broader clinical picture including anthropometric, biochemical, clinical and dietary data, with consideration given to hereditary and lifestyle factors to guide and measure improvements in physical health [20].

Further supporting dietetic involvement in CBT-E is a recent analysis which showed that 60% of AN treatment manuals contain nutrition and food-related information that is not substantiated by current evidence [27]. In CBT-E, the mental health professional is responsible for treating dietary restraint [4], an approach to eating characterised by intention to restrict, delayed eating, food avoidance and dietary rules [28]. Dietitians are specifically trained to address nutrition misinformation and misconceptions about body weight and shape regulation [29] and, as such, have greater nutritional knowledge than mental health professionals [30, 31]. Furthermore, dietitians are skilled in nutrition counselling which has been shown to improve dietary intake and quality [32]. Involving a dietitian in CBT-E may increase treatment efficacy because they are equipped to target malnutrition as well as dietary restraint in an evidence-based manner.

## Proposal

With this rationale we propose a novel practice strategy; Interprofessional Enhanced Cognitive Behaviour Therapy (CBT-IE). CBT-IE maintains the structure and content of CBT-E but involves a dietitian-mental health professional dyad to deliver treatment; delegating interventions related to malnutrition, including dietary restriction and low body weight, as well as dietary restraint, to a dietitian (see Table 1; with detailed overview in Additional file 1). The dietitian will use principles of Medical Nutrition Therapy and nutrition counselling to facilitate improvements in dietary intake. Additionally, the dietitian will complete training in CBT-E and engage in ongoing supervision relevant to eating disorders. The mental health professional and dietitian will access shared clinical notes and conduct case consultations to facilitate ICP. Consent for sharing information among professionals, including shared clinical notes and interprofessional case consultations, will be sought from patients prior to commencing treatment. Case consultations are an opportunity for the dietitian and mental health professional to discuss clinical impressions, progress to date, barriers to change, the formulation and any proposed modifications and future treatment plans. These are held after the initial assessment and again after sessions 7, 20, 30, and the final session. CBT-IE considers the complex interaction between early behaviour change, therapeutic alliance and treatment outcomes, whereby therapeutic alliance positively affects early behaviour change and improves treatment outcomes [33]. Although early behaviour change is completed with the dietitian, involving the mental health professional in the assessment and joint sessions and positioning CBT-IE as truly interprofessional promotes therapeutic alliance among all team members.

**Table 1:** Brief overview of Interprofessional Enhanced Cognitive Behaviour Therapy

Assessment (Sessions A^1^ and A^2^)	The assessment stage of Interprofessional Enhanced Cognitive Behaviour Therapy (CBT-IE) involves an assessment session with the mental health professional (A^1^) and an assessment session with the dietitian (A^2^) The mental health professional’s CBT-IE assessment session is conducted as per ‘The Initial Evaluation Interview’ detailed in Christopher Fairburn’s (14) book ‘Cognitive behavior therapy and eating disorders’ (henceforth referred to as ‘The CBT-E manual’) The dietitian’s CBT-IE assessment session is conducted as per ‘The Initial Session’ of the CBT-E manual, additionally incorporating a standard dietetic assessment An interprofessional case consultation is held after A^1^ and A^2^ are completed
Stage one (Sessions 1–7)	Stage one is focused on gaining a shared understanding of the person’s eating problem, providing education about the impact of the eating disorder, and helping the individual improve their nutritional intake The dietitian delivers stage one of CBT-IE, adapted as per the ‘Underweight and Undereating’ chapter of the CBT-E manual. In addition to CBT-E content, the dietitian will deliver individualised nutritional interventions consistent with Medical Nutrition Therapy for malnutrition and eating disorders Sessions with the mental health professional are conducted in stage one if acute and severe psychological concerns are identified, or where a comorbid mental health concern is the primary barrier to completing stage one tasks
Stage two (Session 8 to maximum 9)	Stage two is a brief transitional stage involving one interprofessional case consultation followed by one or two joint sessions with the dietitian, mental health professional and the patient where progress is reviewed, and plans are made for stage three
Stage three (Sessions 9 or 10 through to maximum 36 sessions)	Stage three is focused on the processes that maintain the person’s eating problem Sessions are delivered by the dietitian and mental health professional in a modular or alternating format at varied frequency based on patient need The dietitian delivers modules ‘Underweight and Undereating’ (where still relevant after stages one and two) and ‘Dietary Restraint, ‘Dietary Rules and Controlling Eating’ as per the CBT-E manual The mental health professional delivers modules ‘Shape Concern, Shape Checking, Feeling Fat and Mindsets’ and ‘Events, Mood and Eating’ as per the CBT-E manual The dietitian delivers optional modules to address comorbid nutrition diagnoses, nutrition for physical activity, and nutrition for weight maintenance if relevant to the patient The mental health professional delivers optional modules to address ‘Clinical Perfectionism’, ‘Core Low Self-Esteem’, and ‘Interpersonal Relationships’ as per the CBT-E manual if relevant to the patient An interprofessional case consultation between the dietitian and mental health professional is held after sessions 20 and 30 Where considered feasible and necessary by treating clinicians, the mental health professional and dietitian will hold joint sessions with the patient
Stage four (The final 4–6 of maximum 40 sessions)	Stage four is concerned with ending treatment well The mental health professional addresses concerns about ending treatment, discusses strategies to ensure psychological progress is maintained, and makes plans to minimise risk of lapse/relapse relating to these issues The dietitian discusses strategies to ensure progress is maintained with regards to dietary change, and makes plans to minimise risk of lapse/relapse relating to these issues The final session is a joint session between the dietitian, mental health professional and the patient An interprofessional case consultation between the dietitian and mental health professional is held after the final session

Establishing ICP between dietitians and mental health professionals in the treatment of AN is not without obstacles. Historically, AN has been treated as a psychiatric condition and involving dietitians in CBT-IE deviates from the established norm which may cause resistance to change among eating disorder professionals. For instance, dietitians may be perceived as inadequately trained in the underlying cognitive processes, emotional components, and principles of psychological treatment due to the discipline’s comparative focus on physiology, biochemistry, nutrition counselling and Medical Nutrition Therapy rather than psychological assessment, diagnosis, and evidence-based psychological interventions. We alleviate these concerns by ensuring that the mental health professional conducts the assessment and is responsible for developing a formulation within the traditional CBT-E framework and delivering key cognitive components of CBT-E. Importantly, the CBT-IE model promotes regular case consultations between professionals and is delivered by teams competent in ICP. CBT-IE offers an opportunity for mental health professionals and dietitians to work together to deliver evidence-based eating disorder treatment which concurrently addresses malnutrition in accordance with evidence-based practice. Additional barriers to the application of ICP between mental health professionals and dietitians is the paucity of evidence-based models of dietetic treatment for AN, which hinders role clarification. CBT-IE manages this through retaining evidence based-content while delegating delivery of nutrition-related aspects to a dietitian. Real world challenges to CBT-IE’s implementation might include establishing training opportunities for collaborative practice as well as logistical concerns, including practitioners located at different clinics without shared practice management software or means to complete joint sessions, although innovations in technology can be used to overcome these issues.

There is a critical need for more effective treatments for AN. Involving dietitians in treatment to directly target malnutrition and dietary restraint is likely important; yet their role remains poorly demarcated in evidence-based treatments despite recommendations for interprofessional approaches within clinical practice guidelines. CBT-IE unites the fields of dietetics and psychology and creates an opportunity to explore mechanisms for ICP between clinicians; a core tenet of AN treatment which has been poorly studied. Since dietitians commonly have shorter waitlists than mental health professionals involving a dietitian may support more timely treatment which has been shown to decrease dropout [34]. Moreover, delegating sessions to a dietitian may offer cost benefits to patients as dietitians typically incur a lower fee for service than mental health professionals. Finally, involvement of a dietitian in CBT-IE may alleviate the workload demand on mental health professionals as sole treatment providers.

Initial research steps will focus on pilot trials, exploring the feasibility of the design of CBT-IE and its acceptability to patients and clinicians, as well as preliminary investigations of efficacy. Training and ongoing supervision of clinicians will ensure adherence to the CBT-IE framework in early studies. Preliminary investigations will consider the impact on clinical features of eating disorder recovery including weight, eating disorder psychopathology and quality of life as well as dietary intake, diet quality, relationship with food, measures of ICP and treatment cost. Collecting data on clinical features relating to diet and ICP is essential, to gather evidence regarding the efficacy of dietetic and interprofessional treatment in eating disorders. Data will be obtained from participants at baseline, mid-treatment, at the end of treatment and post-treatment, and exit surveys obtained from any participants who do not complete the treatment. Like most eating disorder research, recruitment and retention of a sufficient number of participants will likely represent a significant challenge, as will variations in clinician treatment style and availability and motivation of clinicians to champion this novel practice. Future research will also consider the role of general practitioners and psychiatrists alongside CBT-IE, as well as the impact of CBT-IE in other eating disorder presentations where malnutrition may occur such as Bulimia Nervosa. Gathering high quality evidence of the effectiveness of CBT-IE in the treatment of AN will take several years but it is hoped that the inclusion of targeted nutrition intervention and principles of ICP will enhance treatment outcomes for individuals experiencing AN.

## Conclusion

Given AN is characterised by a complex interplay of psychological, nutritional and physiological factors, interventions which recruit clinicians from disciplines of both psychology and dietetics have the potential to improve treatment outcomes. With appropriate empirical foundation and justification, CBT-IE may represent a promising, novel and truly collaborative adaptation to outpatient treatment for AN.

## Supplementary Information

**Additional file 1.** Overview of Interprofessional Enhanced Cognitive Behaviour Therapy (CBT-IE) and interprofessional case consultations.

## Data Availability

Not applicable.
