# Supplementary material for: Collaborative dietetic and psychological care in Interprofessional Enhanced Cognitive Behaviour Therapy for adults with Anorexia Nervosa: a novel treatment approach
Source: J Eat Disord. 2023 Feb 27;11:31. doi: 10.1186/s40337-023-00743-w (PMC9972764; doi:10.1186/s40337-023-00743-w)
Supplement: Supplementary file 1 — Additional file 1. Overview of Interprofessional Enhanced Cognitive Behaviour Therapy (CBT-IE) and interprofessional case consultations. [file 40337_2023_743_MOESM1_ESM.docx]

**Additional file 1:**

**Overview of Interprofessional Enhanced Cognitive Behaviour Therapy (CBT-IE) and interprofessional case consultations**

**Table 1: Overview of Interprofessional Enhanced Cognitive Behaviour Therapy (CBT-IE)**

|  | **Mental Health Professional** | **Dietitian** |
| --- | --- | --- |
| **Assessment**  **- Sessions A^1^ and A^2^**  - Shared clinical notes throughout treatment | **Session A^1^**   1. Conduct ‘The Initial Evaluation Interview’  - As per Christopher Fairburn’s (2008) book Cognitive behavior therapy and eating disorders (henceforth referred to as the CBT-E manual)  1. Explain what treatment will involve 2. Jointly create formulation of the eating problem 3. Recommend regular medical monitoring | **Session A^2^**   1. Conduct ‘The Initial Session’ as per the CBT-E manual 2. Complete standard dietetic assessment as part of the initial session 3. Establish self-monitoring 4. Ensure the patient has commenced regular medical monitoring |
|  | **Interprofessional case consultation (Table 2.0)** | |
| **Stage One**  - **Sessions 1 – 7**  - Weekly sessions  - In-session weighing with dietitian  - Optional appointments with mental health professional* | *Sessions with the mental health professional are conducted in stage one if acute and severe psychological concerns are identified | **Session 1**   1. Initiate in-session weighing 2. Review self-monitoring 3. Set the agenda 4. Work through the agenda (Topics include attitude toward treatment, the formulation and its implications, education about weight checking, education about weight, weight goals and weight change in treatment as per the CBT-E manual) 5. Provide personalised education about the effects of being underweight and/or rapid weight loss if relevant   **Sessions 2-7**   1. Educate the patient about the eating problem 2. Establish regular eating 3. Deliver education regarding weight science 4. Discuss nutritional and physiological principles relevant to regular eating and weight restoration 5. Deliver individualised education regarding clinical symptoms and relationship to undernutrition 6. Deliver individualised nutrition interventions which address clinical symptoms 7. Support nutrition interventions to facilitate .25-1kg weight gain per week 8. Discuss pros and cons of change 9. Address the patient’s style of eating 10. Address purging 11. Address excessive exercise 12. Involve significant others 13. Invite the patient to raise topics for discussion at the upcoming interprofessional case consultation and collaborative session/s |
| **Stage Two**  - **Session 8 to maximum 9**  - A transitional stage where progress is reviewed, and plans are made for stage three  - 1 interprofessional case consultation | **Interprofessional case consultation** | |
|  | **Session 8 to maximum 9**  Mental health professional and dietitian* will discuss outcomes of the interprofessional case consultation, review the formulation, and design stage three with the participant including whether to use focused or broad form CBT-E. Refer to CBT-E manual for recommended session content. | **Session 8 to maximum 9**  *****Although joint sessions are preferred, where this is not practical the mental health professional should independently conduct stage two sessions without the dietitian including whether to use focused or broad form CBT-E. Refer to CBT-E manual for recommended session content. |
| **Stage Three**  - **Sessions 9 or 10 to maximum 36**  - Sessions are delivered by the dietitian and mental health professional in a modular or alternating format at varied frequency based on patient need  - Sessions will range from weekly to fortnightly  - In-session weighing at each session with the mental health professional and dietitian using calibrated scales  - 2 interprofessional case consultations at sessions 20 and 30 | 1. Mental health professional to address Shape Concern, Shape Checking, Feeling Fat and Mindsets. Refer to CBT-E manual for recommended session content | 1. Dietitian to address Underweight and Undereating. The dietitian will use Medical Nutrition Therapy principles for malnutrition and related consequences to support .25-1kg weight gain per week and improved physiological parameters |
|  | **Interprofessional case consultation** | |
|  | 1. Mental health professional to address Events, Moods and Eating. Refer to CBT-E manual for recommended session content | 1. Dietitian to address Dietary Restraint, Dietary Rules and Controlling Eating. Refer to CBT-E manual for recommended session content. The dietitian will provide individualised nutrition science education relevant to the dietary restraint, rules and controlling eating. |
|  | **Interprofessional case consultation** | |
|  | 1. Mental health professional to address Clinical Perfectionism, Low Self-Esteem, and Interpersonal Issues if relevant to the client. Refer to CBT-E manual for recommended session content | 1. Dietitian to address comorbid nutrition diagnoses, nutrition for physical activity, and nutrition for weight maintenance if relevant to the patient |
| **Stage Four**  **- Comprises the final 4 to 6 sessions of treatment to a maximum of 40 sessions**  **-** Focused on ending treatment well | Mental health professional to address concerns about ending treatment, discuss strategies to ensure progress is maintained with regards to overevaluation of shape and weight, event-triggered changes in eating and mindsets, as well as discuss a plan to minimise risk of lapse/relapse in these areas. Mental health professional and dietitian to collaboratively conduct the final session with the patient. | Dietitian to discuss strategies to ensure progress is maintained with regards to regularity, adequacy, and dietary restraint, as well as phase out self-monitoring and in-session weighing. The dietitian will discuss a plan to minimise risk of lapse/relapse in dietary regularity, adequacy, and dietary restraint. Mental health professional and dietitian to collaboratively conduct the final session with the patient. |
|  | **Interprofessional case consultation**  The final interprofessional case consultation is an opportunity to reflect on treatment and hone processes for future service provision. | |

**Table 2.0: Interprofessional Case Consultations**

| Interprofessional Case Consultations to occur after assessment, session 7, 20, 30 and the final session |
| --- |
| **For discussion (Mental Health Professional and Dietitian):**   1. Clinical impression 2. Progress to date  - Weight restoration   - Discuss if weight trend is progressing as expected   - Discuss specific barriers/facilitators to weight restoration   - Dietary intake and quality   - Discuss overall intake of energy, macronutrient and micronutrient intake compared to individual requirements   - Discuss strategies to improve overall intake of energy, macronutrient, and micronutrient intake - Eating disorder cognitions and behaviours   - Discuss current eating disorder thoughts and behaviours   - Discuss specific factors which are known to increase/decrease eating disorder thoughts and behaviours - Other   - Relevant medical parameters   - Engagement in treatment   - Therapeutic relationship   - Comorbid psychiatric or physical illnesses   - Environmental stressors   - Physical activity levels  1. Barriers to change  - Factors relating to weight restoration, dietary intake and quality, eating disorder cognitions and behaviours, and other issues may represent barriers to change - Discuss interprofessional strategies to address barriers to change  1. Formulation and proposed changes  - Extended formulation may include maintaining factors such as core low self-esteem and perfectionism or additional eating disorder behaviours  1. Treatment plan  - Decisions to be made regarding input from mental health professional in stage one and session frequency and structure of stage three |

**References:**

Fairburn, C. (2008). Cognitive behavior therapy and eating disorders. *Cognitive behavior therapy and eating disorders*. Guilford Press.
